# Neural Network Adaptive Inverse Control of Flexible Joint Space Manipulator Considering the Influence of Gravity

**DOI:** 10.3390/s24216942

**Published:** 2024-10-29

**Authors:** Shaoqing Li, Lingcong Meng, Kai Fang, Fucai Liu

**Affiliations:** 1 School of Mechanical and Electrical Engineering, Zhangjiakou Vocational and Technical College, Zhangjiakou 075000, China; lishaoqing123@zjkptc.edu.cn; 2 Key Laboratory of Industrial Computer Control Engineering of Hebei Province, Yanshan University, Qinhuangdao 066004, China; ysu_mlc@163.com (L.M.); fangkai@stumail.ysu.edu.cn (K.F.)

**Keywords:** flexible joint, space manipulator, gravity, RBF neural network, inverse control

## Abstract

With the aim of correcting the problem of trajectory tracking control of a flexible joint space manipulator in environments with different gravity, a neural network adaptive inverse control algorithm based on singular perturbation theory is proposed to resist the disturbance caused by system uncertainty. Firstly, the dynamic model of a flexible joint space manipulator with the influence of gravity is established, and then the system is divided into a fast subsystem and a slow subsystem using singular perturbation theory. The velocity feedback control rate is designed for the fast subsystem to suppress the elastic vibration caused by the joint flexibility. For the slow subsystem, the uncertain term and known term are separated by the inverse control algorithm, where the uncertain term is approximated online by the RBF neural network, and the robust control rate is designed to compensate for the approximation error. The simulation results show that the control method can not only effectively reduce the high-frequency vibration caused by the flexible joint but also resist the system disturbance so that a good track control effect is achieved.

## 1. Introduction

The space manipulator will likely play a very important role in future space activities. In particular, during the construction process of the space station in orbit, the space robot arm can replace the astronaut regarding the assembly and maintenance of the module. Therefore, the positioning accuracy of the robot arm is particularly important in the process of unit assembly. Regarding the improvement of the positioning accuracy of the space manipulator, there are many problems to be considered, but one important point is the influence of gravity release on control performance. For the control system, the dynamic model of the space manipulator from the ground to space will change due to weightlessness, which will also change the operational performance, as well as control accuracy.

Of course, the problem of a floating base and the vibration caused by weight loss or being lightweight should be considered in the practical application process, which affects the positioning accuracy of the space manipulator. Different methods and means can be used to deal with different problems, such as the trajectory tracking problem of the manipulator regarding the floating base, and the trajectory tracking and vibration suppression problem regarding joint flexibility. This paper mainly studies the problem of flexibility and gravity changes from the perspective of control and proposes a neural network adaptive inverse controller. The effectiveness of the algorithm is verified by simulation experiments, which provides a new idea for improving the positioning accuracy of the space manipulator.

In general, the harmonic reducer and planetary gear are adopted as driving parts of the space manipulator and have advantages such as a large reduction ratio, a compact structure, and high transmission efficiency. However, at the same time, they will also bring joint flexibility to the space manipulator, which will lead to lag, nonlinear coupling, high frequency resonance, and other control problems [1]. Research shows that if the joint flexibility of the system is ignored, the control accuracy and system stability of the manipulator will be greatly affected [2]. Therefore, it is necessary to study the control of flexible joint space machinery.

In recent years, researchers have conducted a lot of simulation research on the control problems of the flexible joint space manipulator, such as low tracking accuracy, slow convergence speed, and poor stability. In order to make the flexible joint space manipulator satisfy certain accuracy requirements, Wang L et al. [3] proposed an adaptive controller using radial basis function neural networks to handle unknown uncertainties. Bai X J [4] designed an iterative learning control method, which improved the robustness and stability of the whole system. Zhang L et al. [5] proposed an adaptive fault-tolerant control method. Jing F [6] described an adaptive nonsingular terminal sliding mode (ANTSM) controller based on an extended state observer (ESO) to ensure that tracking errors converge to a small neighborhood of zero. However, the design and tuning of the ESO is relatively complicated, which may affect the dynamic characteristics of the system and lead to a decrease in the accuracy of the observation error. Wei Z [7] proposed an improved radial basis function (RBF) neural network combined with the fuzzy backward step method to identify and suppress random vibration during the operation of the flexible manipulator. The Lyapunov function and control law are designed. However, the training process of the RBF neural network may be affected by the amount and quality of data, and insufficient training may lead to an insufficient generalization ability of the network, thus affecting the vibration suppression effect.

The flexible joint space manipulator must be adjusted on the ground before performing the space task, but the current science and technology, such as flume, suspension, air flotation, and other space microgravity simulation means, cannot completely eliminate the impact of gravity. The gravity change will alter the kinematic and dynamic models of the flexible joint space manipulator, affect the tracking accuracy of the manipulator, and cause an imbalance in the stability of the system. Therefore, in the ground installation and adjustment stage, the influence of gravity on the manipulator system cannot be ignored. Liu F C [8] and others proposed an adaptive robust control method based on singular perturbation theory under the condition of considering the influence of gravity, which can make the flexible joint space manipulator produce a good trajectory tracking control effect. Jia P X [9] and others proposed a new two-pulse input shaper parameter learning strategy for the problem of residual vibration of the flexible joint manipulator, and this method can effectively reduce the residual vibration of the system without an accurate mathematical model. Dmytriv, V et al. [10] analyzed the dynamic error in the partial motion system of the linkage of the robot manipulator. Some equations of motion are derived from Lagrange class II differential equations of motion. The differential equations of positioning errors are solved analytically by the Euler method. However, there are also stability problems in the numerical solution of the Euler method; when the step size is not chosen properly, it is easy to lead to error accumulation. Kaczmarek, W et al. [11] raised the possibility of reducing robot vibration in selected robot production processes such as spot welding. Although methods to reduce robot vibration can help improve control accuracy, there are some disadvantages that make it more difficult to debug and maintain.

The above control method only considers one situation, either ground gravity or space microgravity, and cannot guarantee the ideal trajectory tracking control accuracy of the flexible joint space manipulator at the same time. In this paper, considering the gravity change and the influence of flexible joints, a neural network adaptive inverse control algorithm based on singular perturbation theory is proposed. Extended singular perturbation theory is the mathematical approach to delve into when small parameters in a differential equation cause the behavior of the solution to change significantly. Based on the traditional singular perturbation theory, the system analysis of multiple small parameters, nonlinear effects, and complex boundary conditions are used to deal with a wider range of complex singular perturbation problems. The theory focuses on the behavior of solutions at different scales, master solutions, and higher-order corrections using asymptotic analysis and multiscale techniques to capture these phenomena. It aims to ensure the accuracy and efficiency of solutions to complex problems. According to the time scale, the system is divided into two subsystems: a fast subsystem and a slow subsystem. Regarding the slow subsystem, the inverse control algorithm is used to align and decompose step by step, and the uncertainty term of the system is derived. Gravity is strong on Earth, and gravity is usually a special consideration. In space, microgravity is small, and the dynamics model of the manipulator needs to be specifically designed to accommodate this environment where gravity is usually treated as an uncertainty term in the system. Then, an RBF neural network is used to approximate the uncertainty on line, and a robust control rate is designed to compensate for the approximation error. The speed of the feedback control rate is designed for the fast subsystem to suppress the elastic vibration caused by the flexible joint.

## 2. Dynamic Model

**Figure 1:**
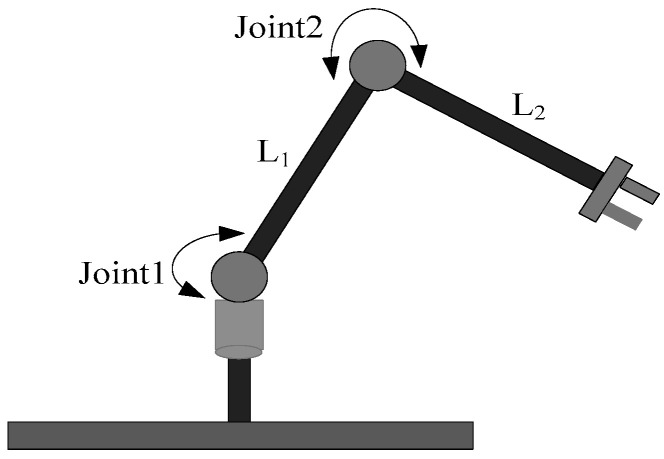
Two-link flexible joint space manipulator.

**Figure 2:**
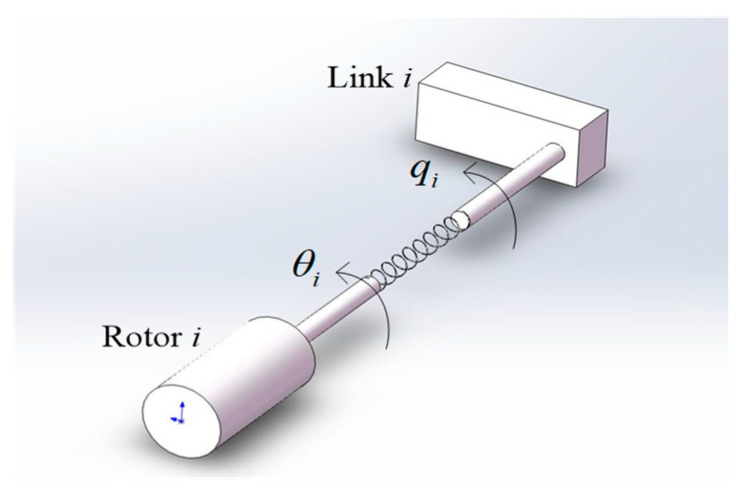
Simplified model of flexible joint.

The flexible joint manipulator is a complex dynamic system consisting of a rigid link and a flexible joint, as shown in Figure 1, which is a schematic diagram of the two-link flexible joint space manipulator model. In this paper, the classical simplified model proposed by Song [12] is adopted. The flexible joint is regarded as a linear torsional spring connected by a motor and the link, and has a constant coefficient. The simplified model is shown in Figure 2.

According to the Euler–Lagrange equation, regarding the joint flexibility, the kinetic energy of the motor rotor is added to the total kinetic energy of the system, and the elastic potential energy of the spring is added to the system potential energy. The dynamic model of the flexible joint space manipulator is as follows:

(1) $$\left\{\begin{array}{l} J_{m}{\ddot{\theta }}_{m}+\tau =\tau_{m}\\ M(q)\ddot{q}+C(q,\dot{q})+G(q)=\tau \\ \tau =K(\theta_{m}-q) \end{array}\right.$$

where $J_{m}$ is the motor moment of inertia matrix, K is the joint stiffness constant matrix, $M(q)\in R^{n\times n}$ is the system inertia matrix, $C(q,\dot{q})\in R^{n\times n}$ is the Coriolis or centrifugal force matrix, $\tau_{m}$ is the motor output drive torque, $\theta_{m}$ is the motor angular displacement vector, and q is the angular displacement vector of the link joint. The dynamics of the link and the dynamics of the actuator are coupled by the vector of elastic torques at joints $K(\theta_{m}-q)$. This is the dynamic model of the flexible joint space manipulator in the microgravity environment in space. In the ground adjustment stage, the influence of gravity is considered, and the dynamic model is as follows:

(2) $$\left\{\begin{array}{l} J_{m}{\ddot{\theta }}_{m}+K(\theta_{m}-q)=\tau_{m}\\ M(q)\ddot{q}+C(q,\dot{q})+G(q)=K(\theta_{m}-q) \end{array}\right.$$

where G is the gravity term, and it changes with the environment. Therefore, the gravity term can be regarded as a part of the uncertainty term of the manipulator system.

## 3. Controller Design

### 3.1. Singular Perturbation Decomposition

The main control idea of singular perturbation theory is to divide the system into fast and slow subsystems according to a time scale [13], and then design the control rate to ensure the stability of the two subsystems. The standard equation of state for the singular perturbation model of the dynamic system is as follows:

(3) $$\left\{\begin{array}{l} \dot{x}=f(t,x,z,\varepsilon )\\ \varepsilon \dot{z}=g(t,x,z,\varepsilon ) \end{array}\right.$$

where $x\in R^{n}$, $z\in R^{n}$ is the system state variable, and ε is the perturbation parameter. When ε=0, 0=g(t,x,z,0), and the system’s quasi-steady-state equation is obtained. When the fast subsystem is designed, the boundary layer subsystem is obtained by neglecting the slow constant.

In this paper, the space manipulator with the flexible joint is decomposed into a fast subsystem and a slow subsystem using singular perturbation. The total control torque of the system is

(4) $$\tau_{m}=\tau_{f}+\tau_{s}$$

where $\tau_{f}$ is the fast control torque of the system. It is used to provide a fast response in case of a sudden change to suppress the elastic vibration produced by the flexible joint. $\tau_{s}$ is the slow control torque, which compensates for the total disturbance of the system and makes the manipulator achieve a high-precision trajectory tracking control effect [14].

From the above system dynamics model, the equation of the joint driving moment τ can be obtained by combining Formulas (1) and (2):

(5) $$J_{m}K^{-1}\ddot{\tau }+\tau =\tau_{f}+\tau_{s}-J_{m}\ddot{q}$$

Introduce parameter ε, take $K=K_{\varepsilon }/\varepsilon^{2}$, where $K_{\varepsilon }$ is the positive definite diagonal matrix; the smaller the ε, the greater the joint stiffness, and select

(6) $$\tau_{f}=-K_{v}\dot{\tau }=-\varepsilon K_{2}\dot{\tau }$$

This formula is the fast control torque of the system, and then the combination of Formulas (5) and (6) can yield

(7) $$\varepsilon^{2}J_{m}\ddot{\tau }+\varepsilon K_{\varepsilon }K_{2}\dot{\tau }+K_{\varepsilon }\tau =K_{\varepsilon }(\tau_{s}-J_{m}\ddot{q})$$

Let ε=0, and the quasi-stationary expression of joint moment is obtained:

(8) $$\tau =\tau_{s}-J_{m}\ddot{q}$$

Combine Equation (8) with Equation (1) to obtain the quasi-steady-state equation of the system:

(9) $$\bar{M}(q)\ddot{q}+C(q,\dot{q})\dot{q}+G(q)=\tau_{s}$$

where $\bar{M}(q)=M(q)+J_{m}$. The quasi-steady-state system has the following dynamic characteristics:

(1) The inertia matrix $\bar{M}(q)\in R^{n\times n}$ is a positive definite and bounded matrix.
(2) $\bar{M}(q)-2C(q,\dot{q})\dot{q}$ is a skew symmetry matrix.

### 3.2. Inverse Controller Design

The basic idea of the inversion design method [15] is to decompose the complex nonlinear system into subsystems that do not exceed the order of the system, and then to design the Lyapunov function and the intermediate virtual control quantity for each respective subsystem, then back to the whole system, and then the design of the whole control rate is completed.

The quasi-steady-state Equation (9) of the system is inversely controlled, and a slow controller is designed. Let $x_{1}=q$, $x_{2}=\dot{q}$, and convert Equation (9) to a state space expression:

(10) $$\left\{\begin{array}{l} {\dot{x}}_{1}=x_{2}\\ {\dot{x}}_{2}={\bar{M}}^{-1}(\tau_{s}-Cx_{2}-G) \end{array}\right.$$

(1) Assuming that $q_{d}$ is the desired joint angle and has a second derivative, the position error $z_{1}$ is defined as

(11) $$z_{1}=x_{1}-q_{d}$$

The virtual control quantity is designed as $\alpha_{1}=-k_{1}z_{1}$, where $k_{1}>0$.

The error $z_{2}$ is defined as

(12) $$z_{2}=x_{2}-\alpha_{1}-{\dot{q}}_{d}=x_{2}+k_{1}z_{1}-{\dot{q}}_{d}$$

(13) $${\dot{z}}_{1}=x_{2}-{\dot{q}}_{d}$$

For the first subsystem in Equation (10), the Lyapunov function is defined as follows:

(14) $$V_{1}=\frac{1}{2}z_{1}^{T}z_{1}$$

(15) $$\begin{array}{l} {\dot{V}}_{1}=z_{1}^{T}{\dot{z}}_{1}=z_{1}^{T}(x_{2}-{\dot{q}}_{d})\\ =z_{1}^{T}(z_{2}-k_{1}z_{1}+{\dot{q}}_{d}-{\dot{q}}_{d})=-k_{1}z_{1}z_{1}^{T}+z_{1}^{T}z_{2} \end{array}$$

If $z_{2}=0$, then ${\dot{V}}_{1}<0$, which means that the first subsystem is stable, so the next step of the design is needed.

(2) The derivation of Equation (13) is as follows:

(16) $$\begin{array}{l} {\dot{z}}_{2}={\dot{x}}_{2}+k_{1}{\dot{z}}_{1}-{\ddot{q}}_{d}\\ ={\bar{M}}^{-1}(\tau_{s}-Cx_{2}-G)+k_{1}{\dot{z}}_{1}-{\ddot{q}}_{d} \end{array}$$

For the second subsystem in Equation (10), the Lyapunov function is used:

(17) $$V_{2}=V_{1}+\frac{1}{2}z_{2}^{T}z_{2}$$

(18) $$\begin{array}{l} {\dot{V}}_{2}={\dot{V}}_{1}+z_{2}^{T}{\dot{z}}_{2}=-k_{1}z_{1}^{T}z_{1}+z_{1}^{T}z_{2}+\\ z_{2}^{T}\left[{\bar{M}}^{-1}(\tau_{s}-Cx_{2}-G)+k_{1}{\dot{z}}_{1}-{\ddot{q}}_{d}\right]\\ =-k_{1}z_{1}^{T}z_{1}+z_{1}^{T}z_{2}+z_{2}^{T}({\bar{M}}^{-1}\tau_{s}+f(x)) \end{array}$$

where f(x) is the uncertainty term of the system and includes the gravity term of the system. In order to make ${\dot{V}}_{2}\leq 0$, the control law is as follows:

(19) $$\tau_{s}=\bar{M}(-k_{2}z_{2}-z_{1}-\hat{f}(x))$$

where $k_{2}>0$, $\hat{f}(x)$ is the estimation of system uncertainty f(x). Next, the uncertainty of the system is approximated using the RBF neural network.

### 3.3. On-Line Approximation of RBF Neural Network

The RBF neural network is a three-layer feedforward network with a single hidden layer [16], and its action function is the Gaussian basis function. Therefore, the RBF neural network is a kind of local approximation network that has fast learning convergence speed and can avoid a local minimum problem. Also, it can approximate any continuous function with high accuracy.

The RBF neural network algorithm can be expressed as follows:

(20) $$\phi_{i}(x)=\exp(-{\left\|x-c_{i}\right\|}^{2}/\sigma_{i}^{2})\;i=1,2,,,m$$

(21) $$f_{n}=W^{T}\varphi (x)=\sum_{1}^{m}w_{i}\phi_{i}(x)$$

where $x\in \Omega_{x}\subset R^{n}$ is the network input signal, $\phi_{i}(x)$ is a radial basis function, m is the number of network nodes, $W\in R^{m}$ is the network weight, $c_{i}$ is the center point, and $\sigma_{i}$ is the base width.

According to the above RBF neural network algorithm, the system uncertainty function in Equation (18) can be approximated online by selecting the appropriate node and base width. The input signal of the neural network is taken as $x={\left[x_{1}\;x_{2}\right]}^{T}$, and its output is

(22) $$\hat{f}(x)={\hat{W}}^{T}\varphi (x)$$

$\hat{f}(x)$ is the estimation of the uncertainty function f(x) of the system, and

(23) $$f(x)=-{\bar{M}}^{-1}(Cx_{2}+G)+k_{1}{\dot{z}}_{1}-{\ddot{q}}_{d}={\left(W^{*}\right)}^{T}\varphi (x)+\varepsilon$$

$W^{*}$ is the ideal neural network weight, and ε is the modeling error of the neural network.

In conclusion, the neural network adaptive inverse control law is designed as follows:

(24) $$\tau_{s}=\bar{M}(-k_{2}z_{2}-z_{1}-{\hat{W}}^{T}\varphi (x)-\delta )$$

δ is a robust control term which is used to compensate for the approximation error ε of the neural network.

The neural network adaptive rate [17] is designed as follows:

(25) $$\dot{\hat{W}}=F\varphi (x)\xi -\eta \left\|\xi \right\|\hat{W}$$

(26) δ=ωsgn(ξ)

where $\xi ={\left[z_{1}z_{2}\right]}^{T}$.

The Lyapunov function is selected as follows:

(27) $$V=\frac{1}{2}z_{1}^{T}z_{1}+\frac{1}{2}z_{2}^{T}z_{2}+\frac{1}{2}tr({\tilde{W}}^{T}F^{-1}\tilde{W})$$

(28) $$\begin{array}{l} \dot{V}=-k_{1}z_{1}^{T}z_{1}+z_{1}^{T}z_{2}+z_{2}^{T}(\tau_{s}-f(x))\\ +tr({\tilde{W}}^{T}F^{-1}\dot{\tilde{W}})=-\xi^{T}K_{t}\xi -z_{2}^{T}(f(x)-\hat{f}(x))\\ -z_{2}^{T}\delta +tr{\tilde{W}}^{T}(-\varphi \xi^{T}+\beta F\left\|\xi \right\|\hat{W}+\varphi \xi^{T})\\ =-\xi^{T}K_{t}\xi -z_{2}^{T}(f(x)-\hat{f}(x))-z_{2}^{T}\delta \\ +\eta F\left\|\xi \right\|tr{\tilde{W}}^{T}(W^{*}-\tilde{W})\leq -\xi^{T}K_{t}\xi +\frac{1}{2}{\left\|z_{1}^{T}\right\|}^{2}\\ +\frac{1}{2}{\left\|z_{2}^{T}\right\|}^{2}+\frac{1}{2}{\left\|\delta \right\|}^{2}+\eta F\left\|\xi \right\|tr{\tilde{W}}^{T}(W^{*}-\tilde{W})\\ \leq -\xi^{T}K_{t}\xi +\eta F\left\|\xi \right\|tr{\tilde{W}}^{T}(W^{*}-\tilde{W}) \end{array}$$

where $\tilde{W}=W^{*}-\hat{W}$ is the weight error. According to the properties of F norm, we can obtain the following:

$$tr\left[{\tilde{x}}^{T}(x-\tilde{x})\right]\leq \left\|\tilde{x}\right\|\left\|x\right\|F-F{\left\|\tilde{x}\right\|}_{F}^{2};\;\mathrm{therefore},\;\;tr{\tilde{W}}^{T}(W^{*}-\tilde{W})\leq {\left\|\tilde{W}\right\|}_{F}{\left\|W^{*}\right\|}_{F}-{\left\|\tilde{W}\right\|}_{F}^{2}.$$

In order to guarantee that $\dot{V}\leq 0$, ${\left\|\tilde{W}\right\|}_{F}>W_{\max}^{*}$ needs to be satisfied.

The general controller of the system is

(29) $$\tau_{m}=-K_{v}\dot{\tau }+\bar{M}(-k_{2}z_{2}-z_{1}-{\hat{W}}^{T}\varphi (x)-\delta )$$

**Figure 3:**
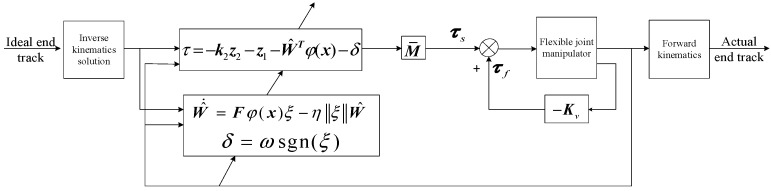
Control block diagram of flexible joint space manipulator system.

The control structure of the flexible joint space manipulator is shown in Figure 3:

## 4. Simulation Results and Analysis

In order to verify the effective control of the neural network adaptive inverse control method for the flexible joint manipulator in environments with different gravity proposed in this paper, the positive direction of anticlockwise movement with a side length of 5 m is selected as the end tracking track for simulation research [18]. Compared with other tracking trajectories, the square trajectory has greater requirements for the controller, and it is easier to observe the end estimation tracking and vibration suppression of the flexible joint space manipulator. Table 1 shows the simulation parameters of two bars of the model.

The model parameters of the flexible joint manipulator system are as follows: K= diag{500}, $J_{m}$ = diag{1}. In the control system design, the choice of the gain parameter $K_{v}$ affects not only the stability of the system but also the steady-state deviation, that is, the approximation performance. In this paper, $K_{v}$ = diag{0.15} is chosen to achieve a good balance. The performance of the robust controller is regulated by the robust term parameter F. Increasing F can effectively suppress the influence of external interference on the system; if F is too small, the system may be more sensitive to interference, thus affecting the tracking performance. In this study, F = diag{10} is set to ensure the robustness of the system. Reasonable settings of the output weighting parameters $k_{1}$ and $k_{2}$ are helpful to improve the approximation ability of the neural network. In this paper, $k_{1}$ is set to 2.5 and $k_{2}$ is set to 30 to optimize the network performance. In addition, the decay factor η is used to control the speed of the weight update. A large learning rate may lead to the instability of the learning process, while a small learning rate may make the learning progress too slow. Therefore, η=0.5 is selected to ensure an appropriate learning speed in this paper. The key parameter ω is then used to counteract uncertainties and external disturbances, and ω=0.1 is set in this study. In the RBF neural network, the selection of the number of nodes has an important impact on the performance of the model; five RBF neural network nodes were chosen in this paper. The center point and base width are the key parameters that affect the learning ability and approximation accuracy of the RBF network. In this study, the center point $c_{i}=0$ was chosen. The basis width controls the expansion degree of the basis function, defines the “flat” or “sharp” degree of the basis function, and chooses the basis width $\sigma_{i}=3.5$ to optimize the network performance.

**Figure 4:**
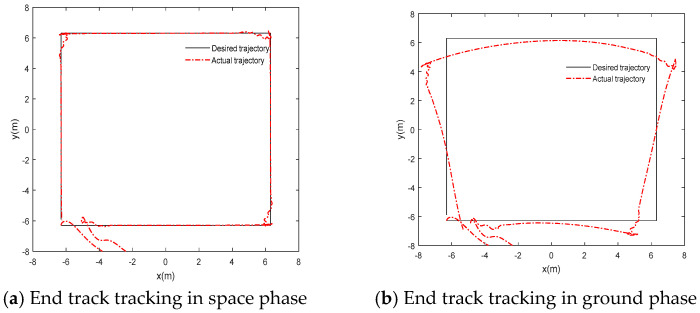
PD control end tracking based on singular perturbation.

**Figure 5:**
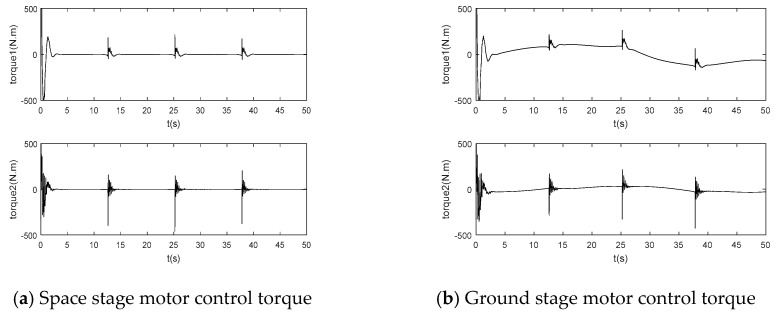
PD control joint driving torque based on singular perturbation.

In order to better show the good control effect of neural network adaptive inverse control, it is compared with the PD control method based on a singular perturbation. The simulation results are shown in Figure 4 and Figure 5:

Based on an analysis of the above simulation results from Figure 4, the flexible joint manipulator can achieve basic trajectory tracking in the space microgravity environment, but the tracking effect is very poor in the ground gravity environment. In Figure 5, the joint driving torque suddenly changes at the corner of the tracked trajectory, and there is serious chattering. If the gravity compensation term is added in the PD controller based on singular perturbation, the flexible joint manipulator can track well in the gravity environment, but it cannot track accurately in the microgravity environment. Therefore, the singular perturbation PD control method cannot meet the control requirements.

**Figure 6:**
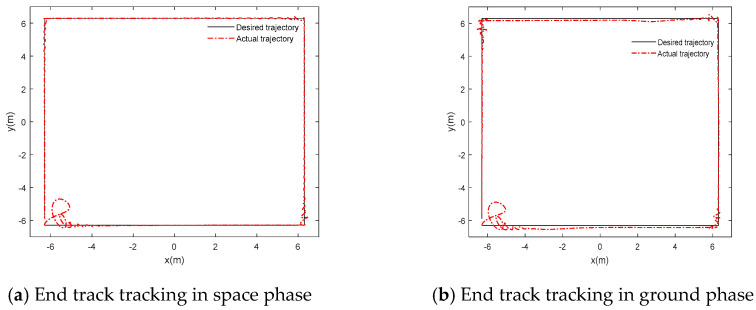
Trajectory tracking of adaptive backstepping control based on singularly perturbed neural network.

The simulation results of trajectory tracking based on adaptive inversion control based on singular perturbation neural network are shown in Figure 6.

**Figure 7:**
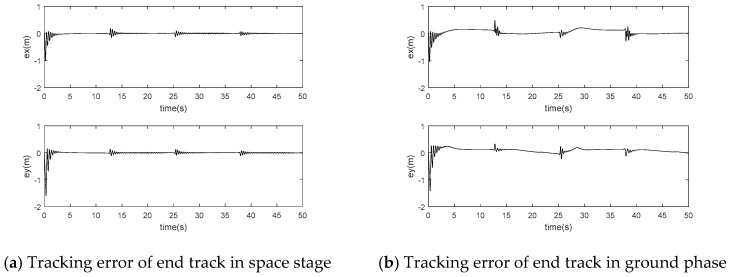
Singular perturbation neural network adaptive inverse control position error.

**Figure 8:**
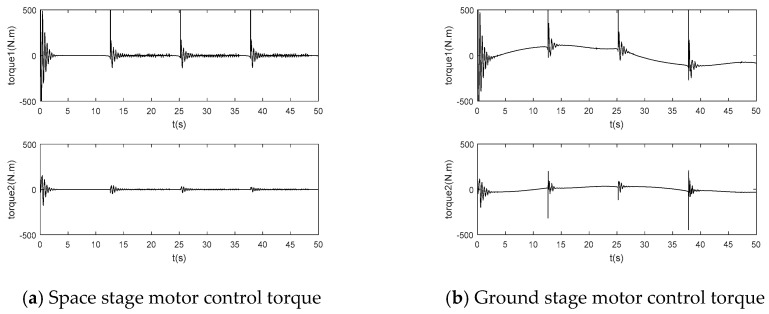
Adaptive inverse control of motor control torque based on singularly perturbed neural network.

**Figure 9:**
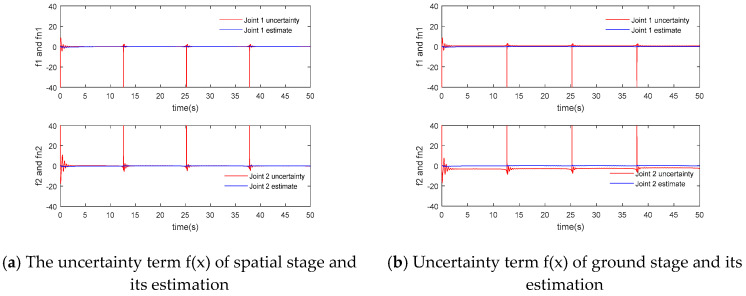
f(x) and its estimation of adaptive backstepping control of singularly perturbed neural networks.

The position error simulation results of adaptive inversion control based on singular perturbation neural network are shown in Figure 7:

The simulation results of joint drive torque based on adaptive inversion control based on singular perturbation neural network are shown in Figure 8:

The simulation results of the adaptive inversion control f(x) based on singular perturbation neural network and its estimation are shown in Figure 9:

According to Figure 6a and Figure 7a, the flexible joint manipulator controlled by neural network adaptive inversion can achieve high-precision trajectory tracking in the space microgravity environment, and the tracking error can quickly converge to zero. In Figure 8a, the joint driving torque changes rapidly with an abrupt change in the track at the square corner so that the error is adjusted to zero. In Figure 9a, the RBF neural network has a good approximation effect on the system uncertainty in the microgravity environment

In the ground installation and adjustment stage, Figure 6b and Figure 7b shows that the flexible joint manipulator still has a good trajectory tracking effect. Figure 8a,b show that the driving torque of the ground phase is larger than that of the space phase due to the influence of gravity. The RBF neural network in Figure 9b still has a good approximation effect for the uncertainty term, including the gravity term, and the flexible joint space manipulator can adapt well to changes in the system disturbance, such as changes in gravity.

In conclusion, the neural network adaptive inverse control algorithm based on singular perturbation theory can ensure that the flexible joint space manipulator can adapt to changes in the gravity environment and can produce a better trajectory tracking effect in both the ground and space stages.

## 5. Conclusions

In this paper, a neural network adaptive inverse control algorithm based on singular perturbation theory is proposed. The uncertainty term of the system is derived by adopting inverse control to reduce the order of the system, and the gravity term is regarded as the uncertainty term. Then, it is approximated using an RBF neural network. An analysis of the above simulation results shows that the controller designed by the neural network adaptive inverse control algorithm can achieve better trajectory tracking and chattering suppression in the ground gravity and space microgravity environments. Compared with the singular perturbation PD control, the neural network adaptive inverse control algorithm does not need the precise model of the system and also can effectively meet the control requirements of the accuracy, robustness, and adaptability of the system.

## Figures and Tables

**Table 1:** Simulation parameters of two-link flexible joint space manipulator.

Link Number	$a_{i}(m)$	$b_{i}(m)$	$m_{i}(kg)$	$I_{i}(kg\cdot m^{2})$
1	2.25	2.25	1.5075	2.5439
2	2.25	2.25	1.5075	2.5439

## Data Availability

The authors reserve the right not to disclose the private dataset used in this study.
